# Exploring Variability in Spinal Anesthesia Levels Achieved During Cesarean Section Deliveries: A Narrative Review

**DOI:** 10.7759/cureus.88429

**Published:** 2025-07-21

**Authors:** Naresh Paliwal, Imran Ahmed Khan

**Affiliations:** 1 Anesthesiology, Dr. Panjabrao Deshmukh Memorial Medical College, Amravati, IND; 2 Community Medicine, KMC Medical College and Hospital, Maharaj Ganj, IND

**Keywords:** bupivacaine, cesarean section, head-down tilt, pregnancy, spinal anesthesia

## Abstract

Spinal anesthesia (SA) is the preferred technique for cesarean section (CS), but variability in sensory block levels poses challenges. This variability becomes particularly prominent in pregnant women, and while the physiological changes of pregnancy are widely recognized contributors, local anesthetic (LA) properties and some technical factors are also responsible for these variations. Literature from 2015 to 2025 was reviewed on PubMed and Google Scholar to identify physiological, technical, and pharmacological factors responsible for spinal block characteristics in parturients. Pregnancy-related changes, e.g., reduced cerebrospinal fluid volume (CSF), technical factors (e.g., interspace misidentification), and drug baricity contribute to variability. A detailed understanding of the physiological changes of pregnancy, fine details of anesthetic technique, and pharmacological properties of medications used is important. Individualized techniques and adopting ultrasound guidance could enhance consistency. Further studies on CSF density and standardized protocols are warranted.

## Introduction and background

Spinal anesthesia (SA) is the most commonly employed technique for cesarean sections (CS) due to its rapid onset, simplicity, and lower rate of complications as compared to general anesthesia (GA) [1-3]. Achieving the ideal sensory block level (typically T4-T6) for CS under SA is crucial for patient comfort and surgical conditions [4,5]. A challenge frequently encountered in clinical practice is the variability in levels achieved among different patients, despite the administration of the same dose and concentration of local anesthetic (LA) during SA. This variability becomes particularly prominent in pregnant women, and while the physiological changes of pregnancy are widely recognized contributors, LA properties and some technical factors are also responsible for these variations [6,7]. This narrative review aims to explore the factors contributing to variability in the sensory block levels achieved during SA for CS, with a focus on the physiological changes of pregnancy, technical considerations, and properties of LAs. It seeks to synthesize existing evidence on these determinants and their clinical implications while also identifying gaps in knowledge to guide future research and improve anesthetic practice in obstetric care.

## Review

This review explores the multifactorial causes of variability in SA levels during CS, focusing on physiological, technical, and pharmacological factors. Relevant studies were identified through PubMed and Google Scholar using keywords such as ‘spinal anesthesia,’ and ‘cesarean section, and synonyms.

Pregnancy-related factors

During pregnancy, several anatomical and physiological changes occur that can influence the spread of intrathecal drugs (Table 1).

**Table 1:** Pregnancy-related changes affecting block characteristics SA: spinal anesthesia; CSF: cerebrospinal fluid; LA: local anesthetics

Pregnancy-related changes	Mechanism of influence on SA spread	Clinical implications
Increased lumbar lordosis	Alters spine curvature, enhances cephalad spread of hyperbaric solutions	Adjust the injection site relative to the lordotic curve
Engorged epidural veins	Compresses the subarachnoid space, enhances spread	Monitor for higher block levels
Reduced CSF volume	Smaller subarachnoid space increases cephalad spread	Use lower LA doses
Lower CSF density	May cause isobaric solutions to act hyperbaric	Consider baricity effects in dosing
Elevated progesterone levels	Increased sensitivity of neuronal tissues to LA	Use lower LA doses

Exaggerated lumbar lordosis (the inward curvature of the lower spine) in late pregnancy can affect the curvature of the subarachnoid space, influencing the gravitational spread of hyperbaric solutions [8]. The gravid uterus compresses the inferior vena cava (IVC), leading to distension of the epidural venous plexus. These engorged veins encroach upon and reduce the volume of both the epidural and subarachnoid spaces. A smaller subarachnoid space means that a given volume of LA will spread more cephalad and produce a higher block. This is a primary reason why pregnant women require lower doses of LA for SA compared to non-pregnant individuals. The total volume of CSF in the subarachnoid space can be reduced due to venous engorgement, further contributing to a more extensive spread of LA. The large uterus increases intra-abdominal pressure, which can influence CSF dynamics and potentially contribute to cephalad spread [9]. CSF density is also slightly lower in term parturients, potentially altering the behavior of isobaric solutions [10]. There is a theoretical possibility that isobaric drugs may exhibit mildly hyperbaric behavior in such settings, thereby contributing to unexpected drug spread and block height.

In addition, the hormonal milieu of pregnancy, particularly elevated progesterone levels, leads to increased sensitivity of neuronal tissues to LA [11]. Although these changes are nearly universal, they do not entirely account for the variability observed in spinal block levels. While the pregnancy-related changes have a marked impact on SA spread, some technical factors need to be considered.

Technical factors

While anatomical and physiological changes of pregnancy are responsible for SA variability, technical factors play an equally critical role (Table 2).

**Table 2:** Technical factors affecting block characteristics L: lumbar

Technical factors	Impact on block characteristics	Mitigation strategies
Interspace misidentification	2–3 spaces higher, leading to high blocks	Use ultrasound guidance, regular training
Injection site (relative to lordotic curve)	Below L3 favors lumbosacral spread; above L3 enhances cephalad spread	Align the dose with the interspace
Patient positioning	With hyperbaric drugs, sitting increases caudal spread, and lateral enhances unequal spread	Immediate supine position
Injection speed	Rapid injection increases spread, slower localizes	Standardize injection over 15–30 seconds

One critical factor is the identification of the correct lumbar interspace for spinal injection. Landmark-guided techniques, still commonly used in clinical settings, often fail to correctly identify the intended space. In one study, only 29% of anesthesiologists accurately located the desired interspace using surface landmarks [12]. Furthermore, landmark-based insertions often result in needle placement two to three spaces higher than anticipated. Even with ultrasound guidance, there remains a margin of error of approximately one interspace, though this is a significant improvement over blind technique [13]. Misidentification of the interspace can lead to either inadequate or unexpectedly high spinal blocks.

Equally important is the relationship between the site of injection and the lumbar lordotic curve. The tip of this curve typically lies at the L3 vertebral level. When the spinal injection is performed below this point, such as at L4-L5, a substantial portion of the drug may distribute preferentially toward the lumbosacral nerve roots. This can result in insufficient cephalad spread and failure to achieve the desired sensory level with hyperbaric bupivacaine. Conversely, injections administered above the curve (L2-L3 or L1-L2) facilitate cephalad spread, allowing adequate block levels to be achieved with smaller volumes. Thoracic segmental spinal anesthesia (TSSA), by virtue, also requires lesser doses [14]. This suggests the importance of aligning the dose with the chosen interspace, particularly in short-statured patients, or alternatively, selecting the interspace based on the intended dose.

Another often-overlooked aspect is the patient's positioning during and immediately after the spinal injection. A sitting position leads to more predictable caudal spread of hyperbaric solutions and can help in identifying the midline, especially in obese patients. However, the patient must be rapidly positioned supine after injection to allow cephalad spread for a surgical block. The lateral decubitus position is also commonly used for CS. The dependent side may receive a slightly denser block, but the overall spread of hyperbaric solutions will be influenced by the table tilt [15].

Frequently, patients are quickly moved from the sitting/lateral to the supine position, sometimes with inadvertent lifting of the pelvis. This can promote excessive cephalad spread of hyperbaric drugs, especially when compounded by the relaxed musculature and anatomical changes in pregnant patients. Immediate repositioning is therefore essential to minimize this risk.

The direction of the needle bevel (if using a cutting needle) or the orifice (pencil-point needle) can theoretically influence the direction of initial drug flow, but its clinical significance on overall block height is often minimal compared to baricity and patient position [16]. While aspiration of clear CSF confirms intrathecal placement, the amount of CSF aspirated or mixed (barbotage) has been debated regarding its effect on spread. Some argue barbotage increases mixing and spread; others find no significant effect [17].

Taller patients generally have a longer spinal column and thus a larger CSF volume, which can dilute the LA and lead to a lower block level for a given dose. On the other hand, shorter patients may experience higher block levels [18]. However, the correlation between height and block level is not always linear or consistent [19, 20]. These conflicting results highlight the need for larger trials. Obese patients often have more epidural fat, which can decrease the volume of the subarachnoid space, potentially leading to a higher block [21, 22]. However, anatomical landmark identification can be challenging in obese patients, sometimes leading to multiple attempts or different insertion sites, which can also influence spread. Individual variations in the shape of the spinal canal, the presence of septa, or the volume of CSF can significantly alter drug spread. Spinal deformities like kyphosis and scoliosis create anatomic distortions, making accurate needle placement difficult and leading to unpredictable spread [23]. Multiple attempts and needle redirections are often required.

Rapid injection can lead to greater turbulence and mixing within the CSF, potentially resulting in a more widespread and higher block. Slower injection may lead to a more localized spread. However, the impact of injection speed is often less significant than baricity and patient position [24]. Warmer solutions may spread slightly more due to reduced viscosity, but this effect is generally minor clinically [25].

Pharmacological factors

The total amount of LA injected is the primary determinant of block height and duration [26]. Higher doses generally result in higher and denser blocks. Drug baricity also plays a role in variability [27]. This is the most crucial factor determining drug spread after intrathecal injection. Baricity refers to the density of the local anesthetic solution relative to CSF. While hyperbaric solutions are preferred for their predictable behavior, isobaric agents may behave differently in pregnant patients due to reduced CSF density. Hyperbaric solutions (most common for CS) are denser than CSF (e.g., bupivacaine with dextrose). These solutions will "sink" with gravity. If injected in the sitting position, they spread caudally. If injected in the supine or Trendelenburg position, they spread cephalad. Isobaric solutions tend to spread more diffusely by convection within the CSF, less predictably influenced by gravity. Opioids (e.g., fentanyl, sufentanil) added to the LA generally do not significantly alter the spread of the LA itself but enhance analgesia and improve block quality [28]. Vasoconstrictors (like epinephrine) can prolong the duration of the block but have minimal impact on initial spread [29]. Clinicians should remain aware of these potential variabilities, particularly when selecting agents for high-risk or complicated cases. Pharmacological impacts are compiled in Table 3.

**Table 3:** Pharmacological factors affecting block characteristics LA: local anesthetic

Pharmacological factor	Impact on block characteristics	Clinical considerations
LA dose	Higher doses increase block height/density	Titrate dose accordingly
Baricity (hyperbaric vs. isobaric)	Hyperbaric sinks with gravity, isobaric diffuses equally	Prefer hyperbaric for predictable spread
Opioids (e.g., fentanyl)	Enhances analgesia, with minimal effect on spread	Add 10–25 μg for improved block quality
Vasoconstrictors (e.g., epinephrine)	Prolongs duration, minimal spread effect	Use cautiously in high-risk cases

Clinical implications

Given the complexity and variability of SA responses, especially in parturients, anesthesiologists must individualize their approach. A variety of neuraxial techniques are now available, offering greater flexibility and control. These include single-shot SA using a single drug or combination drug technique, continuous spinal anesthesia (CSA), which allows titrated dosing, epidural anesthesia alone, and combined spinal-epidural (CSE) anesthesia.

While not a direct physiological or pharmacological factor, the experience of the anesthesiologists in consistently performing the technique, optimizing patient positioning, and accurately identifying landmarks can indirectly influence the consistency and predictability of block levels [30].

The use of adjuvants such as intrathecal opioids or alpha-2 agonists for block enhancement is especially useful in high-risk patients and in cases expected to last a longer duration. In cases with severe cardiac disease, morbid obesity, or preeclampsia, hemodynamic stability and precise control over block height are imperative. The ability to titrate or combine neuraxial approaches offers significant advantages in such cases. In resource-limited settings, lack of ultrasound access may exacerbate variability, necessitating alternative strategies like standardized training.

Although the prospect of high SA is concerning, it is usually manageable with early recognition and supportive measures [31]. One of the earliest and most important clinical signs is a change in the patient’s voice quality, indicating an ascending block. Continuous communication with the patient, monitoring of respiratory pattern and hemodynamics, and prompt intervention are essential. Administration of supplemental oxygen, encouraging deep breathing, and judicious use of vasopressors and fluids typically suffice. In most cases, the sensory level begins to recede within 10-15 minutes, and the patient becomes more comfortable. Intubation is rarely needed unless there is evidence of respiratory compromise.

This narrative review has some limitations that should be considered. The synthesis relies on a selective literature search, which may introduce publication bias by overlooking some relevant articles. The review lacks quantitative meta-analysis due to its narrative nature, potentially missing statistical insights. Additionally, the focus on English-language publications may exclude relevant research in other languages, particularly from regions with high CS rates. These limitations highlight the need for systematic reviews or large-scale prospective studies to validate the identified factors and address variability more comprehensively.

## Conclusions

While the physiological changes of pregnancy undoubtedly influence the spread of intrathecal anesthetic agents, they do not fully explain the variability in levels achieved during SA for CS. Technical factors, such as interspace identification, the vertebral level relative to the lordotic curve, patient positioning, and drug baricity, are equally, if not more, influential. A detailed understanding of these elements, combined with an individualized approach to neuraxial anesthesia, is essential for achieving predictable and safe outcomes in CS. Further studies are needed to quantify CSF density changes in pregnancy or evaluate ultrasound-guided SA in routine practice.
